# A Salt-Responsive *PvHAK12* from *Paspalum vaginatum* Negatively Regulates Salt Tolerance in Transgenic *Arabidopsis thaliana*

**DOI:** 10.3390/ijms27073029

**Published:** 2026-03-26

**Authors:** Ying Zhao, Risheng Huang, Huapeng Zhou, Yuxin Chen, Mengtong Dai, Chuanqi Zhao, Siyu Ran, Fengyuan Liu, Xiangwang Xu, Minjie Wang, Zhenfei Guo, Haifan Shi

**Affiliations:** 1 College of Grassland Science, Nanjing Agricultural University, Nanjing 210095, China; 2 Key Laboratory of Bio-Resource and Eco-Environment of Ministry of Education, College of Life Sciences, Sichuan University, Chengdu 610064, China

**Keywords:** *Paspalum vaginatum*, PvHAK12, ion homeostasis, antioxidant defense system, salt tolerance

## Abstract

Soil salinization has become a major global constraint threatening ecosystem stability and agricultural production. As a prominent salt-tolerant turfgrass, *Paspalum vaginatum* (seashore paspalum) serves as an excellent material for exploring salt tolerance mechanisms. In this study, *PvHAK12*, a high-affinity K^+^ transporter (HAK) family gene isolated from seashore paspalum, was functionally characterized. PvHAK12 encodes a 788 amino acid protein with 13 transmembrane domains, belonging to the plasma membrane-localized ion transporters. It exhibits high sequence conservation with other HAK transporters and is predominantly expressed in roots and stems, with distinct tissue- and time-specific induction under salt stress. Yeast complementation assays revealed that PvHAK12 has no obvious K^+^ transport capacity but may mediate Na^+^ transport. Overexpression of *PvHAK12* in *Arabidopsis thaliana* significantly reduced salt tolerance at germination, seedling and rosette stages, as reflected by lower germination rate, fresh weight, survival rate, the maximum quantum yield of photosystem II (*Fv*/*Fm*) value and chlorophyll content, accompanied by higher ion leakage. Under salt stress, transgenic plants accumulated more Na^+^ and less K^+^, leading to an elevated Na^+^/K^+^ ratio. Moreover, transgenic lines displayed weaker antioxidant enzyme activities and higher reactive oxygen species (ROS) accumulation. Transcript analysis further demonstrated that *PvHAK12* overexpression suppressed the induction of multiple ion-transport and stress-responsive genes under salt conditions. These results indicate that *PvHAK12* negatively regulates plant salt tolerance by disrupting ion homeostasis, antioxidant capacity and stress-related gene expression.

## 1. Introduction

Soil salinization severely threatens ecosystem stability and agricultural productivity [1]. Under saline conditions, plants are subject to multiple detrimental effects, including repressed growth and development, perturbed metabolic homeostasis, and disrupted ionic balance [2]. To cope with salt stress, plants have evolved multiple adaptive mechanisms during long-term evolution, such as ion homeostasis maintenance, osmotic adjustment, antioxidant defense system activation, and hormonal regulation [3]. Specifically, ionic and redox homeostasis regulations are pivotal for enhancing plant salt tolerance, as they can alleviate ionic toxicity and oxidative damage caused by salt stress [4,5].

The K^+^ transporter (KT)/high-affinity K^+^ transporter (HAK)/K^+^ uptake protein (KUP) family plays a key and irreplaceable role in regulating K^+^ uptake and long-distance translocation in plants [6]. Notably, some HAK transporters are capable of mediating the uptake of both K^+^ and Na^+^, while others lack K^+^ transport activity and exclusively function in Na^+^ uptake. The function of transporting K^+^ and Na^+^ is closely related to plant growth and salt stress adaptation [7]. Up to now, this family has been extensively studied and characterized in various model plants and major crops, providing valuable target genes for crop genetic improvement in salt tolerance. For example, OsHAK5 and OsHAK16 have identical functions under salt stress. Either of the two genes enhances K^+^ absorption and root-to-shoot translocation, thereby elevating the shoot K^+^/Na^+^ ratio and improving salt tolerance in rice [8,9]. Under salt stress, OsHAK12 exhibits higher Na^+^ transport activity than K^+^, while OsHAK12 may reduce Na^+^ accumulation in the shoot by absorbing Na^+^ from the xylem vessels, thereby enhancing salt tolerance [10]. ZmHAK4 mediates Na^+^ rather than K^+^ uptake, reducing Na^+^ content in xylem sap and inhibiting Na^+^ transport from roots to shoots, thereby enhancing salt tolerance in maize [11]. SlHAK20 transports Na^+^ and K^+^ and regulates Na^+^ and K^+^ homeostasis under salt stress conditions. A variation in the coding sequence of SlHAK20 was found to be the causative variant associated with Na^+^/K^+^ ratio and confer salt tolerance in tomato [12]. Compared with the extensive research on model plants and major crops, the functional characterization of KT/HAK/KUP family members in halophyte plants remains relatively limited. *Casuarina equisetifolia* CeqHAK6 and CeqHAK11 improved their ability to tolerate salt stress by increasing the K^+^/Na^+^ ratio and antioxidant enzyme activities (CAT, POD, and SOD), and decreasing reactive oxygen species (ROS) accumulation [13]. *Hordeum brevisubulatum HbHAK1* facilitates K^+^ retention and contributes to salt tolerance [14]. The specific promoter of *HbHAK2* induces *HbHAK2* expression, improves K^+^ uptake, promotes root architecture, and increases salt tolerance [15].

As an important component of urban ecosystems, turfgrasses are extensively applied in urban greenbelts, lawn landscapes, and sports fields, where they exert vital effects on optimizing the urban ecological environment, offering leisure and recreational spaces, and boosting the esthetic value of landscapes [16]. However, in actual production and management, turfgrasses are often subjected to salt stress originating from multiple sources, such as irrigation with brackish water, over-fertilization, salt spray in coastal regions, and the application of de-icing salts during winter [17]. Exposure to high-salinity environments can significantly hinder the growth and development of turfgrasses, resulting in the degradation of key agronomic characteristics—including turf quality, leaf pigmentation, coverage rate, seed germination potential, root development, and biomass accumulation. These adverse effects not only impair the ecological and recreational functions of turfgrasses but also bring about considerable economic losses [18]. Thus, enhancing the salt tolerance of turfgrasses holds profound practical importance for their sustainable application.

Among stress-tolerant turfgrass species, *Paspalum vaginatum* (seashore paspalum) serves as an ideal model due to its exceptional salt tolerance and publicly available high-quality genome [19], making it well-suited for deciphering the molecular mechanisms underlying salt stress adaptation. In this study, we identified *PvHAK12*, a salt stress-responsive gene in *P. vaginatum*. Transgenic *Arabidopsis thaliana* overexpressing *PvHAK12* exhibited altered Na^+^/K^+^ homeostasis and ROS dynamics, highlighting its role in salt tolerance. The main research goals of this study are to systematically clarify the molecular mechanism of PvHAK12 in regulating Na^+^/K^+^ balance and ROS scavenging under salt stress, explore its functional divergence from homologous HAK genes in model plants and major crops, and provide a novel functional gene resource and theoretical basis for genetic improvement of salt tolerance in turfgrasses and other crops.

## 2. Results

### 2.1. Isolation and Characterization of PvHAK12

A full-length coding sequence (CDS) of *PvHAK12* with a size of 2367 bp (Pavag07G080900.1) was cloned, which encodes a protein with 788 amino acids. A phylogenetic analysis of the high-affinity K^+^ transporter (HAK) family revealed that PvHAK12 clustered closely with OsHAK11 and OsHAK12 (Figure S1A). The TMHMM transmembrane domain prediction showed that PvHAK12 contains 13 transmembrane helices, with posterior probabilities exceeding 0.8 for most domains, consistent with typical plasma membrane-localized ion transporters (Figure S1B). Multiple sequence alignment demonstrated that PvHAK12 shared highly conserved amino acid motifs with functionally characterized HAK transporters, exhibiting 62.69% sequence identity with *Oryza sativa* OsHAK12 and 92.47% identity with *Sorghum bicolor* SbHAK12 (Figure S1C).

### 2.2. Expression Pattern and Subcellular Localization Analysis of PvHAK12

The subcellular localization of PvHAK12 was investigated through the transient overexpression of the PvHAK12-eGFP fusion protein (enhanced green fluorescent protein, eGFP) in *Nicotiana benthamiana* leaves. The observed fluorescence from PvHAK12-eGFP aligned with AtAKT1-mCherry, which was localized in plasma membranes (Figure S2A,B), suggesting potential localization in the plasma membranes, consistent with its transmembrane domains. Spatial expression analysis revealed that *PvHAK12* transcripts were predominantly detected in stems, roots, stolons, and leaves, with significantly lower levels in leaf sheaths (Figure S3A). Given the salt tolerance of *P. vaginatum* we further examined the impact of salt stress on *PvHAK12* expression. Under NaCl stress, *PvHAK12* expression in roots peaked at 12 h and then declined, whereas in leaves it increased continuously over 48 h (Figure S3B,C). Control groups showed consistently low expression in both organs, indicating distinct temporal and tissue-specific responses of *PvHAK12* to salt stress.

### 2.3. PvHAK12 May Mediate Na^+^ Transport in Yeast

**Figure 1:**
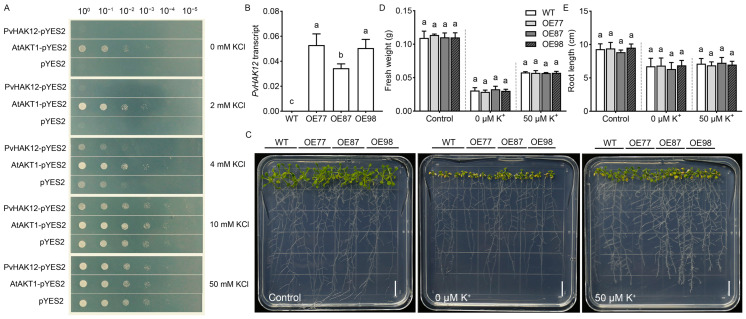
Functional characterization of PvHAK12 in potassium acquisition and plant growth. (**A**) Yeast functional complementation assay. Yeast strains R5421 transformed with PvHAK12-pYES2, AtAKT1-pYES2, or empty pYES2 vector were serially diluted (10^0^ to 10^−5^) and spotted onto arginine phosphate (AP) medium supplemented with 0, 2, 4, 10, or 50 mM KCl. Growth outcomes were documented after a 3-day incubation at 30 °C. (**B**) Transcript levels of *PvHAK12* were analyzed in wild-type (WT, Col-0) and *PvHAK12*-overexpressing transgenic lines (OE77, OE87, OE98). (**C**) Growth phenotypes of *PvHAK12* transgenic *Arabidopsis thaliana* lines (OE77, OE87, OE98) and wild-type plants grown under control, 0 μM K^+^, or 50 μM K^+^ conditions for 14 days. Bar = 1 cm. (**D**,**E**) Quantification of fresh weight (**D**) and root length (**E**) in WT and transgenic lines exposed to control, 0 μM K^+^, or 50 μM K^+^ treatments. Data represent the mean ± SD (n = 3, 15 seedlings per replicate). The same letter above the column indicates no significant difference at *p* > 0.05.

The yeast complementation assay (Figure 1A) revealed that yeast cells transformed with PvHAK12-pYES2 did not exhibit restored growth on media supplemented with 0–50 mM KCl, and their growth was comparable to that of the empty vector control (pYES2). In contrast, the positive control AtAKT1-pYES2 supported robust yeast growth under these low-K^+^ conditions. *PvHAK12* transcripts were detected in three homozygous lines (OE77, OE87 and OE98) compared with the wild-type (WT, Col-0) (Figure 1B). In *Arabidopsis thaliana*, phenotypic analysis showed that under K^+^-sufficient control conditions, the fresh weight and root length of *PvHAK12* transgenic lines (OE77, OE87, OE98) were similar to those of wild-type plants (Figure 1C–E). When grown under K^+^-deficient conditions (0 μM or 50 μM K^+^), no significant differences in fresh weight or root length were detected between the transgenic lines and WT plants (Figure 1C–E). These results suggest that *PvHAK12* overexpression does not enhance K^+^ acquisition or improve tolerance to low-K^+^ stress in plants. These observations indicate that PvHAK12 lacks functional K^+^ transport activity in heterologous systems.

**Figure 2:**
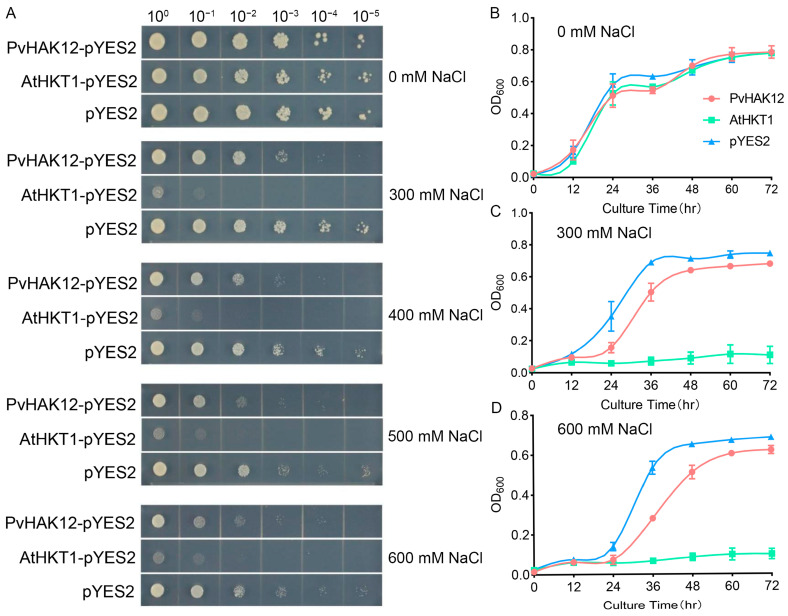
PvHAK12 may mediate Na^+^ transport in yeast. (**A**) Growth comparison of yeast strain BY4741-expressing *PvHAK12*, *AtHKT1*, or harboring the empty vector (pYES2) on solid AP medium with varying concentrations of NaCl (0, 300, 400, 500 and 600 mM). Growth outcomes were documented after a 3-day incubation at 30 °C. (**B**) Yeast cells transformed with the empty vector pYES2 (blue), PvHAK12 (red), or AtHKT1 (green) were cultured in liquid medium supplemented with 0 mM (**B**), 300 mM (**C**), or 600 mM (**D**) NaCl. Cell density was measured as optical density at 600 nm (OD_600_) at 12 h intervals over 72 h. Data represent the mean ± SD of three biological replicates.

The function of PvHAK12 in Na^+^ transport was also tested by yeast complementation, with AtHKT1 as a positive control and empty pYES2 as a negative control. In spot dilution assays, yeast cells transformed with pYES2 showed gradual growth inhibition across serial dilutions on media containing 0, 300, 400, 500, and 600 mM NaCl. In contrast, cells expressing AtHKT1-pYES2 showed more severe growth inhibition at NaCl concentrations of 300 mM and above, which was consistent with previous reports [20], while those expressing PvHAK12-pYES2 exhibited intermediate growth, which was better than AtHKT1 but slightly reduced compared to pYES2 (Figure 2A). Consistent with these results, liquid growth curve analysis confirmed that under 0, 300, and 600 mM NaCl, pYES2 control yeast displayed the highest growth rates and final cell densities (Figure 2B–D). *PvHAK12*-expressing yeast grew more slowly than pYES2 but better than *AtHKT1*-expressing cells, which showed drastically impaired growth under salt stress (Figure 2B–D). Collectively, these observations indicate that *AtHKT1* expression impairs yeast growth under high NaCl conditions, whereas *PvHAK12* expression partially mitigates this growth inhibition, but it does not fully restore growth to the level of the pYES2 control. This suggests that PvHAK12 may contribute to Na^+^ transport in yeast, albeit with a regulatory effect of Na^+^ transport that is distinct from the strong Na^+^ sensitivity conferred by AtHKT1.

### 2.4. Analysis of Salt Tolerance in Transgenic Plants Overexpressing PvHAK12

**Figure 3:**
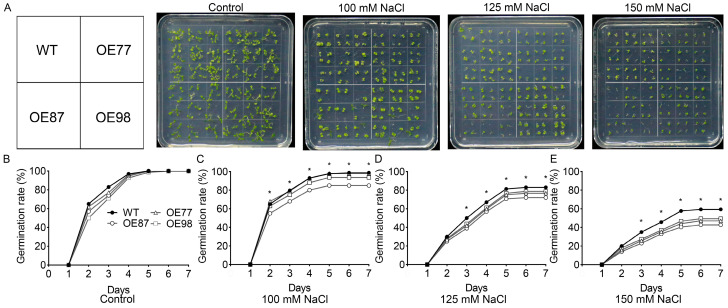
Effect of salt stress on germination rate of *A. thaliana* overexpressing *PvHAK12*. (**A**) Seeds of WT and *PvHAK12* transgenic lines each were horizontally sown on half-strength Murashige and Skoog (1/2 MS) medium containing 0, 100, 125 or 150 mM NaCl. The photography was taken after 7 d of germination. (**B**) Seed germination rate on 1/2 MS medium containing 0 (**B**), 100 (**C**), 125 (**D**) or 150 mM NaCl (**E**) were measured. Data represent the mean ± SD (n = 3, about 45 seeds per replicate). The asterisk indicates significant difference between transgenic lines and the WT at the given time at *p* < 0.05.

To evaluate the role of *PvHAK12* in plant salt stress response, transgenic *A. thaliana* plants overexpressing *PvHAK12* were generated. Plant growth and seed germination are usually used for evaluation of salt tolerance. The seed germination rates of WT and transgenic lines were comparable on half-strength Murashige and Skoog (1/2 MS) medium without NaCl, reaching 98–100% after 7 days (Figure 3A,B). Salt stress reduced seed germination rates in both WT and transgenic lines, and lower levels were observed in transgenic lines than in WT (Figure 3A,C–E). After 7 days of salt stress at 100, 125, and 150 mM NaCl, WT had germination rates of 98%, 83%, and 59%, respectively, while transgenic lines had germination rates of 85–98%, 72–79%, and 42–49%, respectively (Figure 3A,C–E).

**Figure 4:**
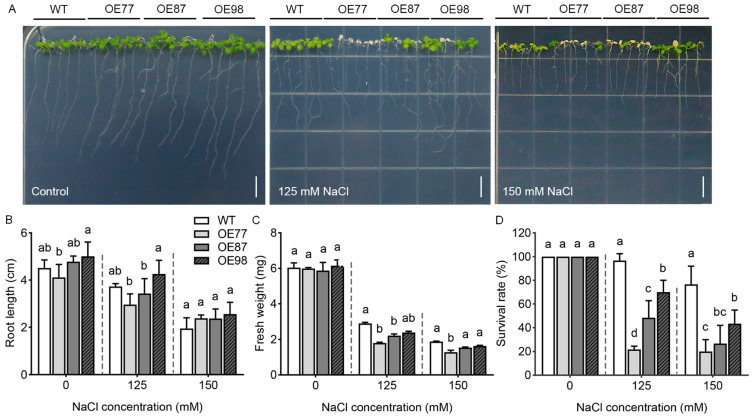
Effect of *PvHAK12* overexpression on plant primary root length, fresh weight, and survival rate in response to salt stress. (**A**) Four-day-old seedlings of WT and *PvHAK12* transgenic lines were transferred to 1/2 MS containing 0, 125 and 150 mM NaCl, and allowed to grow further for 7 days. Bar = 1 cm. (**B**–**D**) Primary root length (**B**), fresh weight (**C**) and survival rate (**D**) of seedlings were measured. At least 30 seedlings were used in each assay. Data represent the mean ± SD (n = 3, >30 seedlings per replicate). Different letters indicate significant differences *p* < 0.05.

Salt tolerance at seedling stage was evaluated based on root length, fresh weight and survival rate. Seedling growth showed no difference between the two genotypes under control conditions. Salinity treatment with 125 or 150 mM NaCl resulted in reduced plant growth, with decreased fresh weight and root length. The transgenic lines showed reduced fresh weight but similar root length relative to the WT (Figure 4A–C). All plant lines maintained a 100% survival rate under control conditions. Under 125 mM or 150 mM NaCl treatment, the survival rate of WT was drastically reduced, while the transgenic lines exhibited significantly lower survival rates (Figure 4D). Collectively, these results demonstrate that overexpression of *PvHAK12* reduces salt tolerance in *A. thaliana* at seedling stage.

**Figure 5:**
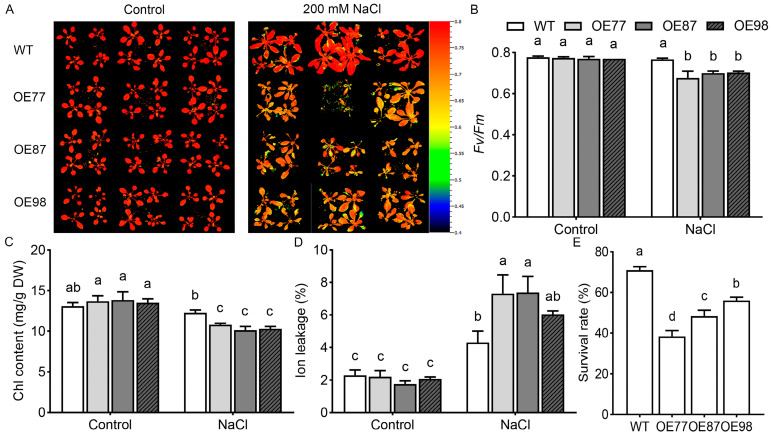
Salt tolerance evaluation of *PvHAK12* transgenic *A. thaliana* lines at the rosette stage. (**A**) The maximum quantum yield of photosystem II (*Fv*/*Fm*) images of leaves from WT and OE lines under control or salt stress. Pseudocolor scale indicates *Fv*/*Fm* values (red = high photosystem II efficiency, blue = low). (**B**) Quantification of *Fv*/*Fm* values from (**A**). (**C**) Chlorophyll content (mg/g dry weight) of WT and *PvHAK12* transgenic lines under control or salt stress. (**D**) Ion leakage rate (%) of WT and *PvHAK12* transgenic lines under control or salt stress, indicating membrane damage. (**E**) Quantitative analysis of survival rates. Data are mean ± SD (n = 3). Different lowercase letters indicate significant differences (*p* < 0.05).

Salt tolerance was further evaluated at the rosette stage. The maximum quantum yield of photosystem II (*Fv*/*Fm*), measured after an appropriate dark-adaptation period, is a widely used parameter for assessing leaf physiological status under stress [21]. Four-week-old plants grown in soil were irrigated with 200 mM NaCl solution for 7 days, and *Fv*/*Fm* was measured. There was no significant difference in *Fv*/*Fm* between transgenic lines and WT under the control condition. *Fv*/*Fm* decreased after salinity treatment, with lower levels in transgenic lines than in WT (Figure 5A,B). Chlorophyll content was measured to assess the impact of salt stress on photosynthetic pigment stability. Under control conditions, there were no significant differences in total chlorophyll content between transgenic lines (OE77, OE87, OE98) and WT plants. Following exposure to 200 mM NaCl stress, chlorophyll content decreased in all genotypes, but transgenic lines retained significantly lower levels of chlorophyll compared with WT (Figure 5C). This observation indicates that the overexpression of *PvHAK12* exacerbates salt-induced chlorophyll degradation, thereby impairing the photosynthetic apparatus and contributing to the reduced salt tolerance in transgenic plants. Ion leakage was used as an index of membrane stability. There were no significant differences in ion leakage between transgenic lines and WT. By contrast, under salt stress, ion leakage was significantly higher in OE lines than in WT, indicating impaired membrane integrity in OE lines upon exposure to salt stress (Figure 5D). The survival rate was measured after the plants were subjected to 16 d of 200 mM NaCl solution. The results showed that transgenic lines had lower survival rates (38–56%) compared with WT (71%) under salt stress (Figure 5E), whereas no significant difference in survival rates was observed between the two under control conditions. Collectively, the above results indicated that overexpression of *PvHAK12* led to decreased salt tolerance in transgenic plants.

### 2.5. Overexpression of PvHAK12 Affects Ion and Reactive Oxygen Species (ROS) Homeostasis Under Salinity Conditions

**Figure 6:**
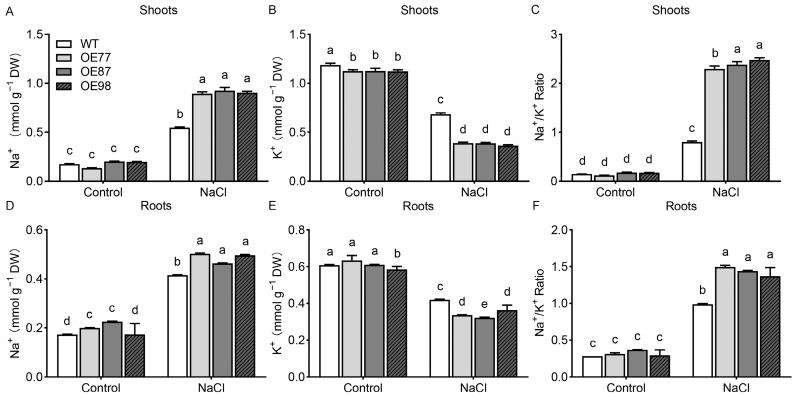
Analysis of Na^+^ and K^+^ concentrations in *PvHAK12* overexpressing lines in comparison with the wild-type after salinity treatment. Four-week-old seedlings were treated for 4 d in 1/2 Hoagland solution containing 50 mM NaCl. Untreated plants were used as control. (**A**) Na^+^ content in shoot. (**B**) K^+^ content in shoot. (**C**) Na^+^/K^+^ ratio in shoot. (**D**) Na^+^ content in root. (**E**) K^+^ content in root. (**F**) Na^+^/K^+^ ratio in root. The present data are mean ± SD (n = 3). The same letter above the column indicates no significant difference at *p* > 0.05. DW, dry weight.

Maintenance of Na^+^ and K^+^ homeostasis is critical for salt stress tolerance. Under control conditions, there were no significant differences in shoot Na^+^ concentrations between the WT and *PvHAK12* overexpressing lines (Figure 6A). In roots, however, the Na^+^ concentrations of WT and OE98 were slightly lower than those of OE77 and OE87 (Figure 6D). For shoot K^+^ concentrations, all transgenic lines exhibited slightly lower levels compared with WT (Figure 6B). In roots, the K^+^ concentration of OE98 was lower than that of WT, OE77, and OE87 (Figure 6E). Under NaCl treatment, Na^+^ concentrations increased significantly in all lines, with transgenic lines accumulating significantly higher Na^+^ in both shoots and roots relative to WT (Figure 6A,D). Although K^+^ concentrations decreased in all plants under salt stress, transgenic lines still maintained significantly lower K^+^ levels compared with WT (Figure 6B,E). These alterations in Na^+^ and K^+^ homeostasis resulted in elevated Na^+^/K^+^ ratios in all genotypes under salinity stress. Notably, *PvHAK12* transgenic lines displayed significantly higher Na^+^/K^+^ ratios compared with WT (Figure 6C,F), which is consistent with their reduced salt tolerance phenotype.

**Figure 7:**
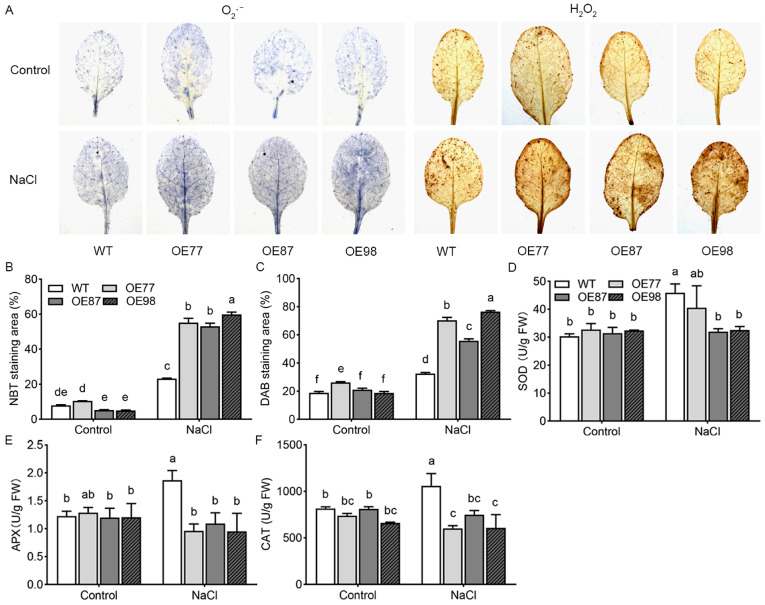
Analysis of reactive oxygen species (ROS) accumulation and antioxidant enzyme activities in *PvHAK12* transgenic lines in comparison with theWT in response to salt stress. (**A**) Four-week-old plants of WT and *PvHAK12* overexpression lines were irrigated with 200 mM NaCl solution for 7 d, while those untreated with NaCl were served as control. NBT and DAB solution was used to detect superoxide free radicals (O_2_^−^) and hydrogen peroxide (H_2_O_2_). Images obtained from NBT (**B**) and DAB (**C**) staining were quantified using ImageJ software. Leaves were sampled for measurements of superoxide dismutase (SOD, (**D**)), ascorbate peroxidase (APX, (**E**)), and catalase (CAT, (**F**)) activities. The present data are means ± SD (n = 3). Different letters indicate significant differences among groups at *p* < 0.05.

Oxidative damage usually occurs when plants are exposed to salt stress. Nitroblue tetrazolium (NBT) and 3,3′-diaminobenzidine (DAB) staining revealed that, compared to control conditions, salt stress induced accumulation of superoxide anion (O_2_·^−^) and hydrogen peroxide (H_2_O_2_) in all genotypes. Salt stress resulted in accumulation of O_2_·^−^ and H_2_O_2_, and higher levels were observed in transgenic lines than in WT (Figure 7A). In order to evaluate the accumulation of ROS, ImageJ software was used to quantify the staining area. Under control conditions, the NBT staining area of WT and OE77 was higher than that of OE87 and OE98, and the DAB staining area of OE77 was higher than that of other lines. After 7 days of 200 mM NaCl treatment, the NBT and DAB staining areas of all lines increased significantly, and the staining area of overexpression lines was significantly higher than that of the WT (Figure 7B,C). Superoxide dismutase (SOD), catalase (CAT) and ascorbate peroxidase (APX) activities showed no difference between WT and transgenic lines under control conditions (Figure 7D–F). Salt stress induced increases in SOD, CAT, and APX activities, with transgenic lines showing lower activities than WT (Figure 7D–F). Salt stress induced the increase of SOD, APX and CAT activities in WT, and the activity of transgenic lines was lower than that of WT. Reduced antioxidant enzyme activities are likely related to the increased ROS and decreased salt tolerance in *PvHAK12* transgenic lines. 

### 2.6. Analysis of Transcripts of the Genes Involved in Ion Homeostasis and Stress Responsive Genes

**Figure 8:**
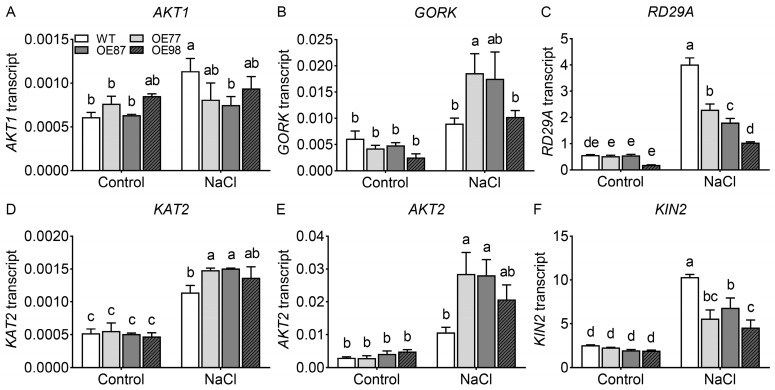
Expression analysis of ion transport and stress-responsive genes in WT and *PvHAK12* transgenic lines under salt stress. (**A**–**F**) Relative expression levels of ion transporter genes *AKT1* (**A**), *GORK* (**B**), *KAT2* (**D**), *AKT2* (**E**) and stress-responsive genes *RD29A* (**C**), *KIN2* (**F**) in WT and OE lines under control conditions or 150 mM NaCl treatment. Transcript abundance was normalized to reference genes, and data are presented as means ± SD (n = 3). Different letters indicate significant differences among groups at *p* < 0.05. The experiment comprised three biological replicates (three independent batches of plant materials cultured under identical conditions). For each biological replicate, three technical replicates were conducted. The data presented are from three technical replicates of one representative biological replicate, as consistent expression patterns were observed across all three biological replicates.

To determine whether the altered Na^+^ and K^+^ homeostasis in *PvHAK12* transgenic plants is associated with changes in the expression of genes involved in ion balance, we examined the transcript levels of ion homeostasis-related genes in WT and transgenic *A. thaliana* before and after salt stress treatment. These genes included K^+^ transporter genes (*KUP4*, *KUP7*, *HAK5*, *HKT1*), K^+^ channel genes (*AKT1*, *AKT2*, *KAT1*, *KAT2*, *GORK*), and the Na^+^/H^+^ antiporter gene *SOS1*. Under control conditions, the transcript levels of these genes were comparable between the WT and all the transgenic lines (OE77, OE87, OE98), with only a few individual lines showing slight differences (Figure 8 and Figure S4). Following salt treatment, the transcript levels of *AKT1*, *KAT2*, *HAK5*, *KUP7*, *SOS1*, and *KUP4* were upregulated in WT. In the three transgenic lines, *AKT1* expression was induced by salt treatment but remained lower than in WT. In contrast, the transcript levels of *KAT2*, and *AKT2* were significantly higher in all three transgenic lines than in WT (Figure 8A,D,E). Except for OE98, the transcription level of *GORK* in the other two transgenic lines was significantly higher than that in WT (Figure 8B). Under salt stress, the transcript level of *KUP7* was increased in WT, OE77, and OE87, but showed no significant change in OE98 (Figure S4C). Under both normal and salt-treated conditions, *HAK5* expression in the OE87 line was higher than in the other lines (Figure S4A). After salt treatment, the transcript levels of *SOS1* and *KUP4* showed no significant change in the OE98 line, but were upregulated in the other lines (Figure S4E,F). The transcript levels of *HKT1* were reduced in all lines under salt stress, with no significant differences detected among them (Figure S4B). Under both normal and salt conditions, *KAT1* transcript levels were similar between WT and the other two transgenic lines, whereas its expression in the OE98 line was significantly lower than in the other lines (Figure S4D). These results indicate that the reduced salt tolerance of *PvHAK12* transgenic lines is associated with the altered expression of K^+^ and Na^+^ transport-related genes *AKT1*, *GORK*, *KAT2*, and *AKT2* under salt stress.

To further investigate whether the reduced salt tolerance in *PvHAK12* transgenic lines was related to the expression of stress-responsive genes, we examined the transcript levels of *RD29A* (Responsive to Desiccation 29A) and *KIN2* (Cold-Responsive 6.6). Under control conditions, the expression of *RD29A* and *KIN2* showed no significant differences between the WT and the three transgenic lines (Figure 8C,F). After salt treatment, both genes were significantly upregulated in WT and transgenic plants, but their transcript levels were markedly lower in transgenic lines than in the WT (Figure 8C,F). This was also in agreement with the reduced salt tolerance phenotype of the transgenic lines. These results indicate that overexpression of *PvHAK12* suppresses the transcriptional induction of genes associated with Na^+^/K^+^ homeostasis and stress adaptation under salt stress, which may contribute to the compromised salt tolerance of transgenic plants.

## 3. Discussion

In this study, a salt-sensitivity-related gene *PvHAK12* was identified from the halophyte *P. vaginatum*. Sequence analysis revealed that PvHAK12 shares high homology with OsHAK12 and SbHAK12. Although PvHAK12 was localized to the plasma membrane, yeast complementation assays (Figure 1A) and heterologous expression in *A. thaliana* (Figure 1C) verified that PvHAK12 is not involved in cytosolic K^+^ transport but in Na^+^ transport, which is consistent with the reported function of its homolog OsHAK12. Most HAK transporters function as K^+^ transporters, yet certain HAK family members are permeable to Na^+^. For example, the heterologous expression of *OsHAK18*, *ZmHAK4*, or *ZmHAK17* failed to complement the growth defect or enhance the growth of K^+^ uptake-deficient yeast under low K^+^ conditions, and instead caused Na^+^ hypersensitivity in the yeast [11,22,23]. This study, along with previous studies, indicated that these HAKs mediate Na^+^ uptake rather than K^+^ transport, which is a unique functional characteristic distinct from most typical HAK transporters.

Notably, our findings demonstrate that PvHAK12 acts as a negative regulator of salt tolerance in *A. thaliana*. Under salt stress conditions, transgenic lines overexpressing *PvHAK12* exhibited a significantly increased shoot Na^+^/K^+^ ratio compared to WT plants (Figure 6C), indicating that PvHAK12 confers salt sensitivity by disrupting cellular Na^+^/K^+^ homeostasis. This regulatory mechanism contrasts sharply with the homologous OsHAK12 in rice, which enhances salt tolerance by elevating the K^+^/Na^+^ ratio via promoting root K^+^ uptake and xylem loading [10]. The divergent functions of PvHAK12 and OsHAK12 may arise from differences in their tissue-specific expression patterns and regulatory networks. In *P. vaginatum*, *PvHAK12* showed high transcript levels in roots and stems, with salt-induced expression peaking at 12 h in roots but remaining elevated in leaves for 48 h (Figure S3B,C). This spatiotemporal expression could lead to Na^+^ accumulation in both roots and shoots. Root expression may enhance Na^+^ uptake or xylem loading, while leaf expression might disrupt mesophyll ion balance, exacerbating photosynthetic damage. In contrast, *OsHAK12* is predominantly expressed in rice roots with minimal shoot expression, functioning to retrieve Na^+^ from xylem vessels and restrict shoot Na^+^ accumulation [10]. Such root-specific localization likely enables OsHAK12 to act as an underground Na^+^ barrier, whereas PvHAK12 may accelerate Na^+^ toxicity in leaves.

Exposure to salinity triggers the rapid generation of reactive oxygen species (ROS) in plants, including hydrogen peroxide (H_2_O_2_), singlet oxygen (^1^O_2_), superoxide anion (O_2_·^−^), and hydroxyl radical (·OH), which arises from metabolic perturbations [24]. In this study, the overexpression of *PvHAK12* in *A. thaliana* led to increased ROS accumulation under salt stress. Simultaneously, under salinity, the activities of key antioxidant enzymes SOD, CAT, and APX were considerably lower in transgenic lines than in WT plants (Figure 7D–F). These results imply that PvHAK12 could have a negative regulatory function in the antioxidant enzyme system under salt stress conditions. Nevertheless, the detailed regulatory mechanisms mediated by PvHAK12 in the antioxidant defense system and ROS accumulation remain unclear. More studies are required to elucidate the detailed molecular events related to its regulatory role.

In Arabidopsis, AKT1, GORK, KUP4 and AKT2 are well-known components involved in K^+^ uptake and transport. AKT1 is an inward rectifier potassium channel that participates in the absorption of K^+^ in roots [25]. GORK functions as an outward-rectifier potassium channel that regulates stomatal closure by mediating K^+^ efflux [26,27]. KAT2 mediates inward K^+^ currents in guard cells and regulates stomatal opening [28]. KAT2 is also highly expressed in the veins phloem, where it contributes to K^+^ loading and long-distance transport [29]. AKT2 participates in the loading and unloading of K^+^ in the cortical cells [30,31]. In this study, the expression patterns of these key K^+^ transport genes were significantly altered in *PvHAK12* transgenic lines under salt stress. Compared with the wild-type, transcript levels of the inward K^+^ channel gene *AKT1* were lower in transgenic lines after salt treatment, which may compromise K^+^ uptake capacity in roots. In contrast, the transcript levels of *GORK*, *KAT2*, and *AKT2* were remarkably higher in transgenic lines. Given that GORK mediates K^+^ efflux during stomatal movement and KAT2 contributes to stomatal opening and K^+^ distribution, the upregulation of these genes likely disrupts intracellular K^+^ homeostasis and ion balance under salt stress. Moreover, the expression of *KUP4*, *KUP7*, *HAK5*, and *SOS1* also exhibited line-specific changes in response to salt, further supporting that overexpression of *PvHAK12* broadly disturbs the transcriptional regulation of the core Na^+^ and K^+^ transport system. Taken together, these expression changes collectively contribute to impaired K^+^ retention, excessive K^+^ loss, and disrupted Na^+^ detoxification in transgenic plants. Our findings suggest that the reduced salt tolerance of *PvHAK12* transgenic lines is closely associated with the imbalanced transcription of major K^+^ and Na^+^ transporter genes, which ultimately disrupts ion homeostasis under saline conditions.

The negative regulatory role of PvHAK12 is consistent with previous reports on HAK transporters. For instance, PvHAK16 from *P. vaginatum* and CsHAK4 from *Camellia sinensis* similarly reduce salt tolerance in transgenic *A. thaliana* by maintaining a lower K^+^/Na^+^ ratio under salinity [32,33]. These findings collectively support functional diversification within the HAK family, where certain members enhance salt tolerance via ion homeostasis, while others act as negative regulators. Furthermore, the link between HAK transporters and salt tolerance is supported by evolutionary studies. Genome-wide association studies in tomato identified a major SlHAK20 variant associated with improved K^+^/Na^+^ homeostasis and salt tolerance, highlighting the role of HAK family members in plant adaptive evolution [12]. Collectively, these observations underscore the complexity of HAK-mediated salt stress responses and emphasize the need for further investigation into the molecular mechanisms underlying PvHAK12 function. Notably, while overexpression of *PvHAK12* in *A. thaliana* has provided preliminary insights into its role in salt tolerance, it is critical to note that these results were obtained from a heterologous system. To gain a more profound understanding of the physiological significance and natural function of PvHAK12 in salt stress responses, future research will concentrate on verifying its function in *P. vaginatum*.

## 4. Materials and Methods

### 4.1. Plant Growth and Treatment

*P. vaginatum* plants were clonally propagated from tillers of the ‘Sea Spray’ cultivar, cultured in a mixture of nutrient-rich soil and perlite (2:1) for two months, and grown in a greenhouse under natural light. In order to analyze the tissue expression pattern of *PvHAK12*, leaf sheaths, leaves, stems, stolons and roots were isolated from plants for RNA extraction. Three biological replicates were set for each sample. To analyze the expression pattern of *PvHAK12* in response to salt stress, *P. vaginatum* was treated with 250 mM NaCl in 1/2 Hoagland nutrient solution, as described in a previous study [34]. Samples were taken at 0, 2, 6, 12, 24 and 48 h for RNA extraction. Three replicates were taken at each time point. The plants without NaCl treatment were used as controls.

Tobacco (*Nicotiana benthamiana*) plants were grown in nutrient-rich soil and vermiculite (1:1 *v*/*v*) mixed soil with a growth chamber at 22 °C. The light was 200 μmol m^−2^ s^−1^ and the photoperiod was 16/8 h (light/dark).

### 4.2. Sequence Analysis

The full-length CDS and amino acid sequence of PvHAK12 and its homologous sequences from *Oryza sativa* and *Sorghum bicolor* were obtained from Phytozome (https://phytozome-next.jgi.doe.gov) accessed on 12 August 2025. Amino acid sequence alignment of PvHAK12 homologs from rice (*Oryza sativa*) and sorghum (*Sorghum bicolor*) was performed using the software DNAMAN v9.0. A minimum-evolution phylogenetic tree was constructed in MEGA11 using the Poisson model, with branch support evaluated from 1000 bootstrap replicates. Accession numbers and species for all included amino acid sequences are detailed in Table S1. Putative transmembrane domains within the PvHAK12 protein sequence were predicted using the TMHMM Server v. 2.0 (https://services.healthtech.dtu.dk/services/TMHMM-2.0/) accessed on 12 August 2025.

### 4.3. Cloning of PvHAK12 Gene

TsingZol Total RNA Extraction Reagent (Qingke, Beijing, China) was used to isolate total RNA from *P. vaginatum* cultivar ‘Sea Spray’. The first-strand cDNA was synthesized using HiScript II Q RT SuperMix for qPCR Kit (Vazyme Biotech, Nanjing, China) according to the manufacturer’s instructions. Primers were designed using Oligo7 software, and the full-length CDS was amplified by PCR using Phanta Max Super-Fidelity DNA Polymerase (Vazyme Biotech, Nanjing, China) and confirmed by sequencing. Primer information is listed in Table S2.

### 4.4. Quantitative Real-Time PCR (qRT-PCR) Analysis

Three independent biological replicates were used, with three plants per replicate. Total RNA was extracted using an TsingZol Total RNA Extraction Reagent (Qingke, Beijing, China). qRT-PCR was conducted on a Thermal Cycler Dice™ Real-Time System (Takara Bio Inc., Shiga, Japan) using ChamQ SYBR qPCR Master Mix (Vazyme Biotech, Nanjing, China) with 30× diluted cDNA templates, per manufacturer protocols. The Clathrin Adaptor Complex Subunit (*CACS*) gene was utilized as an internal reference. In a previous study, the *CACS* gene was identified as the most stable among 12 potential reference genes under salt stress, and its stability was within an acceptable range for cold and drought stresses [35]. *Ubiquitin 10* (*UBQ10*) was used as a reference in *A. thaliana* [36]. The relative expression levels of the genes were calculated using the (2^−ΔΔCt^) method [37].

For analysis of ion and stress responsive genes, seven-day-old *A. thaliana* seedlings germinated on 1/2 MS medium were transferred onto new medium containing 0 or 150 mM NaCl as described previously [38]. WT and transgenic lines of each treatment were harvested after 6 h. Then, total RNA was extracted. The transcript levels of ten ion homeostasis genes *SOS1* (AT2G01980), *GORK* (AT5G37500), *HAK5* (AT4G13420), *KUP4* (AT4G23640), *KUP7* (AT5G09400), *KAT1* (AT5G46240), *KAT2* (AT4G18290), *AKT1* (AT2G26650), *AKT2* (AT4G22200) and *HKT1* (AT4G10310), as well as two stress-responsive genes, *RD29A* (AT5G52310) and *KIN2* (AT5G15970), were evaluated. The specific sequences of all primers used are detailed in Table S2.

### 4.5. Subcellular Localization of PvHAK12 Protein

The *PvHAK12* coding sequence was fused with eGFP at the 5′-end of the pCAMBIA1305 vector driven by the constitutive cauliflower mosaic virus (CaMV) 35S promoter. The pCAMBIA1305-PvHAK12:eGFP plasmid and the plasma membrane localization marker pCAMBIA1305-AtAKT1:mCherry [39] plasmid were co-transformed into one-month-old tobacco leaves using the Agrobacterium-mediated method for subcellular localization. After incubation at 25 °C for 2 days, the tobacco leaf epidermis was visualized using a confocal laser scanning microscope (Carl Zeiss SAS, Jena, Thuringia, Germany). Co-localization analysis was performed using ImageJ v1.54m software based on fluorescence intensity.

### 4.6. Functional Complementation Assays in Yeast

For yeast functional assays, the coding sequences of *PvHAK12*, *AtAKT1*, and *AtHKT1* were individually cloned into the pYES2 vector. The recombinant plasmids and empty pYES2 vector were separately transformed into either the K^+^ uptake-deficient mutant R5421 (for K^+^ complementation) or the salt-sensitive strain BY4741 (for salt tolerance). Transformants were pre-cultured in AP liquid medium to an OD_600_ of 1.0, then subjected to 10-fold serial dilutions (10^0^ to 10^−5^). Aliquots of 2.5 μL were spotted onto AP agar plates (2% galactose) supplemented with either 0, 2, 4, 10, or 50 mM KCl (for R5421) or 0, 300, 400, 500, or 600 mM NaCl (for BY4741). Plates were incubated at 30 °C for 3 days, and growth was assessed. For growth curve analysis, BY4741 transformants were pre-cultured overnight in AP liquid medium, then transferred to AP liquid medium with 0, 300, or 600 mM NaCl, at an initial OD_600_ of ~0.1. Cultures were shaken at 200 rpm, and OD_600_ was measured every 12 h over 3 days. All experiments were performed in triplicate.

### 4.7. Generation of Transgenic Plants

The obtained CDS of *PvHAK12* was inserted into the downstream of the 35S promoter of the CaMV in the pFGC5941 vector using the BamHI and NcoI restriction sites, and the Basta resistance gene was used as a screening marker. The primers used to amplify the *PvHAK12* fragment are shown in Table S2. The constructed plasmid was introduced into the *Agrobacterium strain* EHA105. *A. thaliana* transformation was carried out using the inflorescence dipping technique [40]. The seeds were sown on plant nutrient agar plates supplemented with 10 mg/L Basta and identified by polymerase chain reaction (PCR); the transgenic plants were screened to T3 generation for the experiment.

### 4.8. Low-K^+^ Stress Treatment in A. thaliana

After seed surface disinfection, the seeds germinated on 1/2 MS medium. After 5 days of germination, the seedlings were transferred to new 1/2 MS medium (20 mM K^+^) or low-K^+^ (LK) medium containing 0 or 50 μM KCl for germination at 22 °C. The medium contained 0.8% (*w*/*v*) agar and 3% (*w*/*v*) sucrose. The LK medium used in this study was previously described in [41]. The final K^+^ concentration in LK medium was adjusted by adding KCl. Finally, the main root length and fresh weight were measured.

### 4.9. Salt Tolerance Assays

The surface of *A. thaliana* seeds was disinfected with 8% (*v*/*v*) sodium hypochlorite for 10 min, and then rinsed with sterile water for 5 times. The WT and transgenic *A. thaliana* seeds were sterilized and placed on a 1/2 MS medium containing 0, 100, 125 or 150 mM NaCl, and the culture dish was placed at 4 °C for 2 days. Subsequently, they were cultured at 22 °C for 7 days, and the number of germinated seeds was recorded to calculate the germination rate. The treatment was performed in three biological replicates, with one replicate consisting of about 45 seeds.

For determination of root elongation and survival rate during the seedling stage, the germinated seeds on 1/2 MS medium were transferred onto new medium containing 0, 125 or 150 mM NaCl to allow for seedling growth for 7 d vertically in a growth chamber. Three biological replicates were set up for each treatment, each replicate containing more than 30 seedlings. The survival rate was calculated as the ratio of surviving plants to the total number of plants. Death was characterized by albino leaves. The root length was determined by ImageJ v1.54m.

For salt stress treatment at the rosette stage, 7-day-old seedlings cultured on 1/2 MS medium were transplanted into nutrient soil and acclimatized in a growth chamber for 3 weeks. Subsequently, plants were irrigated with 200 mM NaCl solution. After 7 days of treatment, the maximum quantum yield of photosystem II (*Fv*/*Fm*) was measured using a Pulse-Modulated Fluorometer (FluorCAM 700 MF, PSI, Brno, Czech Republic) according to the manufacturer’s instructions. Leaf samples were collected to determine relative ion leakage and chlorophyll content. In addition, in situ staining for superoxide anion (O_2_·^−^) and hydrogen peroxide (H_2_O_2_), as well as assays for antioxidant enzyme activities, were also performed. These measurements were conducted with three biological replicates, with one replicate consisting of four plants. The survival rate was calculated after a 16-day treatment with 200 mM NaCl, followed by a 5-day recovery period. It was determined as the ratio of surviving plants to the total number of plants, with three biological replicates set up for this index, each consisting of 12 plants. Death was characterized by the yellowing and drying of stems and leaves.

### 4.10. Chlorophyll Content

For both control and salt-treated groups, 0.1 g leaf samples were collected. Leaf tissues were immersed in 10 mL of 95% ethanol and incubated in darkness at room temperature with shaking at 150 rpm for 24 h until complete bleaching. Absorbance at 663 nm (A663) and 645 nm (A645) was measured using a UV-visible spectrophotometer. Chlorophyll concentrations were calculated using the following formulas: Chlorophyll a (mg/L) = 12.72 × A663 − 2.69 × A645; Chlorophyll b (mg/L) = 22.88 × A645 − 4.68 × A663; Total chlorophyll (mg/L) = Chlorophyll a + Chlorophyll b.

### 4.11. Leaf Relative Ion Leakage

Three *A. thaliana* leaves of uniform size from the same leaf position were selected and placed into a glass test tube. Then, 6 mL of deionized water was added, and the conductivity of water was recorded as E0. The tubes were shaken horizontally at 100 rpm for 4 h at room temperature, and the conductivity was then measured using a conductivity meter (DDSJ-307F, INESA Scientific Instrument Co., Ltd., Shanghai, China) and recorded as E1, with the conductivity of deionized water alone (blank control) simultaneously measured and recorded as E2. The test tubes were then placed in boiling water for 20 min, followed by horizontal shaking at 200 rpm for 1 h. After cooling to room temperature, the conductivity was measured and recorded as E3. Relative ion leakage was calculated using the following formula: Relative ion leakage = (E1 − E0)/(E3 − E2) × 100%.

### 4.12. Determination of ROS and Antioxidant Enzymes Activities

In situ staining for O_2_·^−^ and H_2_O_2_ was performed following a previously described method [42]. Leaves were immersed in a 1 mg/mL nitroblue tetrazolium (NBT) solution for 1 h (for O_2_·^−^ staining) or in a 1 mg/mL 3, 3′-diaminobenzidine (DAB) solution for 24 h (for H_2_O_2_ staining). After staining, the leaves were soaked in 95% ethanol until completely decolorized. The leaves were observed and photographed under a microscope. Images from NBT and DAB staining were quantified using ImageJ v1.54m software.

Then, 0.15 g of fresh leaf tissue was taken and ground in a pre-cooled mortar with 1.5 mL of extraction buffer (Solarbio, Beijing, China), which is used for extracting superoxide dismutase (SOD), catalase (CAT), and ascorbate peroxidase (APX), to obtain into a homogenate. The homogenate was centrifuged at 8000× *g* for 10 min, and the supernatant was collected and kept on ice for subsequent assays. The subsequent measurements were performed according to the instructions of the assay kits (Solarbio, Beijing, China), and the activities were calculated.

### 4.13. Determinations of Na^+^ and K^+^

Surface-sterilized *A. thaliana* seeds were sown on 1/2 MS medium. After one week, seedlings were transferred to 1/2 Hoagland nutrient solution for three weeks of cultivation. Subsequently, the *A. thaliana* seedlings were subjected to salt stress by transferring them to a 1/2 Hoagland nutrient solution containing 50 mM NaCl. Plants were harvested at two time points: before stress treatment (as the control) and on the 4th day after salt treatment. Plant samples were rinsed thoroughly with deionized water and dried at 80 °C. A 0.5 g aliquot of dried tissue was digested in a microwave (ETHOS ONE, Milestone, Sorisole, BG, Italy) at 160 °C for 45 min. Na^+^ and K^+^ contents were quantified using inductively coupled plasma optical emission spectrometry (ICP-OES, Optima 8000; PerkinElmer, Shelton, CT, USA). All experiments were performed with three independent biological replicates.

### 4.14. Statistical Analysis

All data were subjected to two-way analysis of variance (two-way ANOVA) using GraphPad Prism software (Version 8.0.1). Significant differences were calculated based on Tukey’s multiple comparisons test at *p* < 0.05.

## 5. Conclusions

In summary, this study systematically characterized PvHAK12, a salt stress-responsive HAK family member isolated from the salt-tolerant turfgrass *Paspalum vaginatum*. Phylogenetic analysis, transmembrane domain prediction, multiple sequence alignment, and subcellular localization confirmed it is a typical plasma membrane-localized ion transporter with high sequence similarity to functionally characterized HAK transporters (especially *Sorghum bicolor* SbHAK12) and widely expressed in *P. vaginatum* tissues with distinct salt-induced expression profiles in roots and leaves. Functional characterization via yeast complementation assays showed that PvHAK12 lacks K^+^ transport activity but mediates Na^+^ transport, and its overexpression in *Arabidopsis thaliana* significantly reduced salt tolerance at seed germination, seedling, and rosette stages, as reflected by decreased germination rates, fresh weight, survival rate, the maximum quantum yield of photosystem II (*Fv*/*Fm*) value, and chlorophyll content, as well as increased ion leakage. Mechanistically, *PvHAK12* overexpression disturbed Na^+^/K^+^ homeostasis (elevating Na^+^ accumulation, reducing K^+^ content, and increasing Na^+^/K^+^ ratios), suppressed antioxidant enzyme (SOD, CAT, APX) accumulation, increased ROS levels, and dysregulated the expression of ion transport-related (*AKT1*, *GORK*, *KAT2*, *AKT2*) and stress-responsive (*RD29A*, *KIN2*) genes under salt stress. Collectively, our findings indicate PvHAK12 functions as a Na^+^ transporter that negatively regulates plant salt tolerance by disrupting ion and ROS homeostasis and key stress-related gene expression, enriching understanding of HAK family functional divergence in halophytic turfgrasses, providing a theoretical basis for exploring *P. vaginatum* salt tolerance mechanisms, and offering a potential target gene for turfgrass and crop salt tolerance genetic improvement.

## Data Availability

The original contributions presented in this study are included in the article/Supplementary Materials. Further inquiries can be directed to the corresponding author(s).

## Supplementary Materials

The following supporting information can be downloaded at https://www.mdpi.com/article/10.3390/ijms27073029/s1.
