# Supplementary material for: A Salt-Responsive PvHAK12 from Paspalum vaginatum Negatively Regulates Salt Tolerance in Transgenic Arabidopsis thaliana
Source: Int J Mol Sci. 2026 Mar 26;27(7):3029. doi: 10.3390/ijms27073029 (PMC13072838; doi:10.3390/ijms27073029)
Supplement: Supplementary file 1 [file ijms-27-03029-s001.zip › Supplementary figures.pdf]

Figure S1

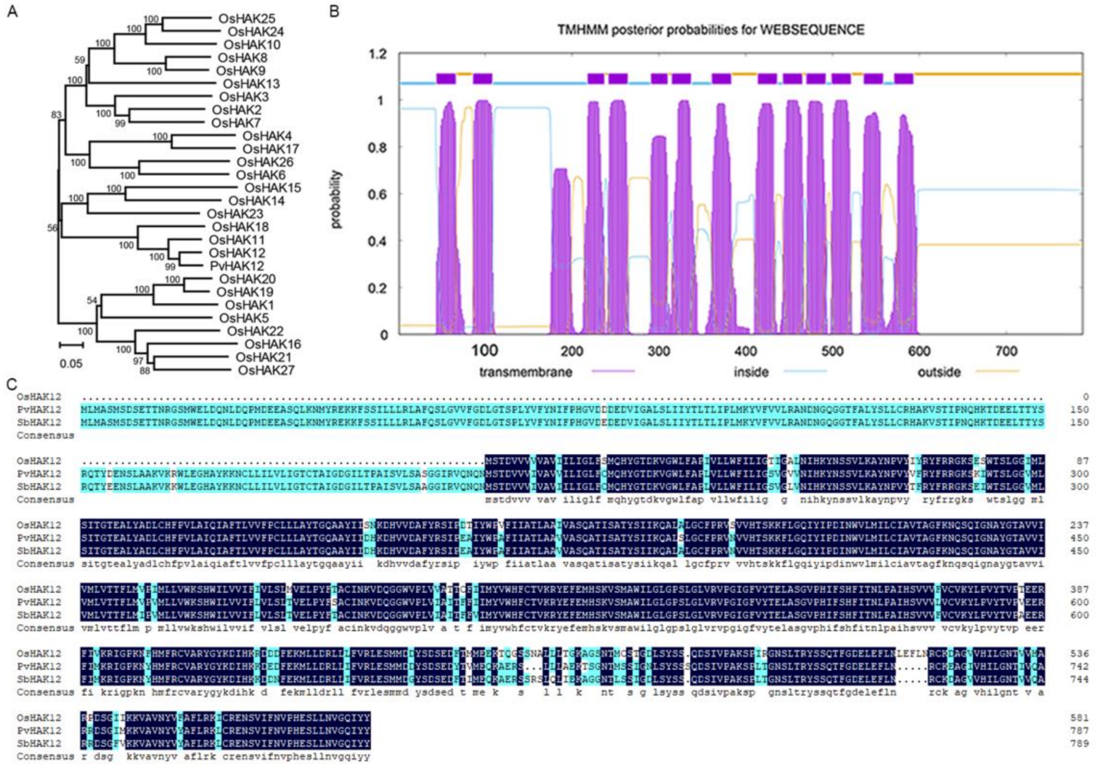

Figure S1. Molecular characteristics of PvHAK12

(A) Phylogenetic analysis of PvHAK12 with HAK family from *Oryza sativa*. The tree was constructed using the neighbor-joining method with MEGA11 software. (B) TMHMM Server v. 2.0 was used to analyze the protein sequence and predict the transmembrane domain of PvHAK12. (C) Alignment of PvHAK12 protein sequence and related protein sequences from *O. sativa* and *Sorghum bicolor*.

Figure S2

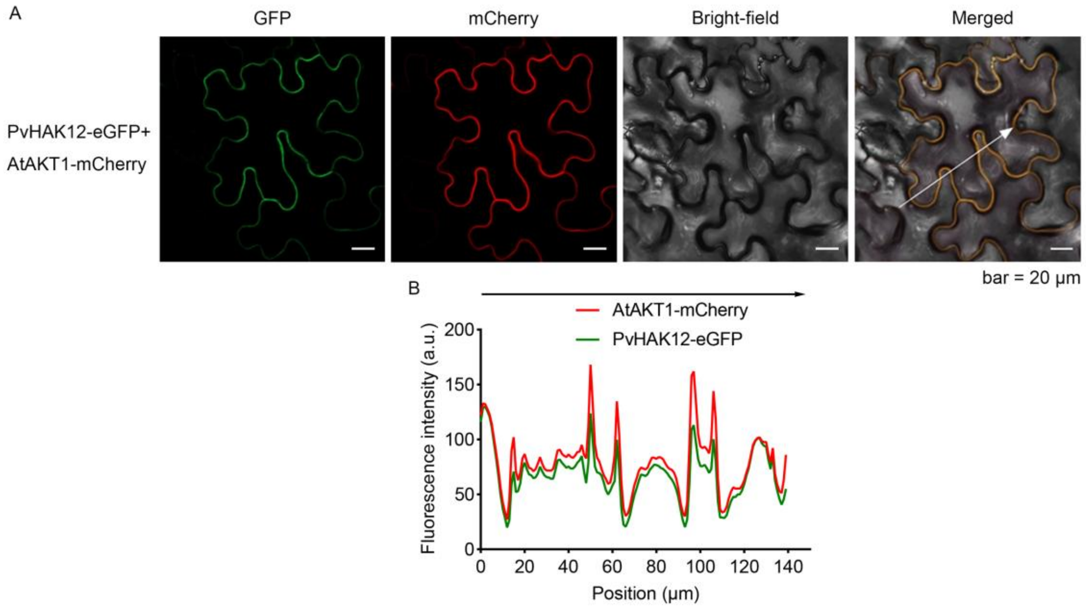

Figure S2. Analysis of subcellular localization of PvHAK12

(A) Visualization of PvHAK12-eGFP (green fluorescent protein) fusion protein and AtAKT1-mCherry (red fluorescent protein) using confocal laser scanning microscopy. The panel displays fluorescence images of PvHAK12-eGFP (green), AtAKT1-mCherry (red), a merged image of both fluorescences, and a corresponding bright-field image. Bar = 20 μm. (B) Colocalization analysis between PvHAK12 and AtAKT1 in a tobacco leaf cell, as indicated by the white arrow in (A), utilizing fluorescence intensity measurements (in arbitrary units) and Image J software.

Figure S3

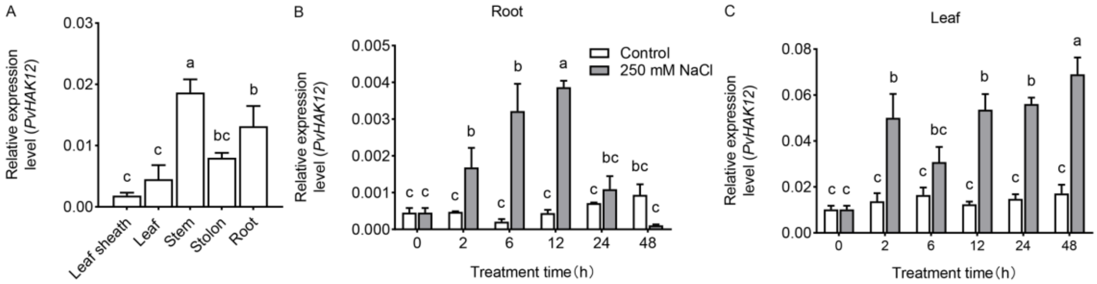

Figure S3. Analysis of *PvHAK12* expression patterns

(A) Analysis of *PvHAK12* expression in plant tissues of seashore paspalum including leaf sheath, leaf, stem, stolon and root. Analysis of *PvHAK12* expression in root (B) and leaf (C) in response to salt stress. Data are mean ± SD (n = 3). Different letters indicate significant differences $P < 0.05$.

Figure S4

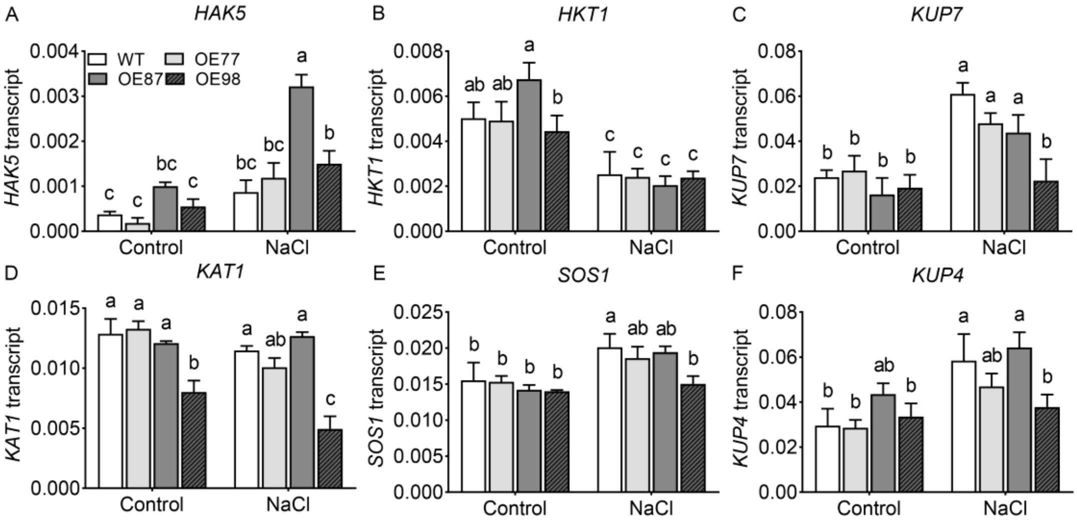

Figure S4. The expression level of ion homeostasis-related genes

Transcript levels of (A) *HAK5*, (B) *HKT1*, (C) *KUP7*, (D) *KAT1*, (E) *SOS1*, (F) *KUP4* of seven-day-old *A. thaliana* seedlings treated with 150 mM NaCl for 6 h. Data are mean ± SD (n = 3). The same letter above the column indicates no significant difference at $P < 0.05$.
